# Species–environment relationships of fish and map‐based variables in small boreal streams: Linkages with climate change and bioassessment

**DOI:** 10.1002/ece3.7848

**Published:** 2021-06-29

**Authors:** Tapio Sutela, Teppo Vehanen, Pekka Jounela, Jukka Aroviita

**Affiliations:** ^1^ Natural Resources Institute Finland (Luke) Oulu Finland; ^2^ Natural Resources Institute Finland Helsinki Finland; ^3^ Natural Resources Institute Finland Turku Finland; ^4^ Finnish Environment Institute Freshwater Centre Oulu Finland

**Keywords:** boreal, climate change, land use, logistic regression, self‐organizing map, small stream

## Abstract

Species–environment relationships were studied between the occurrence of 13 fish and lamprey species and 9 mainly map‐based environmental variables of Finnish boreal small streams. A self‐organizing map (SOM) analysis showed strong relationships between the fish species and environmental variables in a single model (explained variance 55.9%). Besides basic environmental variables such as altitude, catchment size, and mean temperature, land cover variables were also explored. A logistic regression analysis indicated that the occurrence probability of brown trout, *Salmo trutta* L., decreased with an increasing percentage of peatland ditch drainage in the upper catchment. Ninespine stickleback, *Pungitius pungitius* (L.), and three‐spined stickleback, *Gasterosteus aculeatus* L., seemed to benefit from urban areas in the upper catchment. Discovered relationships between fish species occurrence and land‐use attributes are encouraging for the development of fish‐based bioassessment for small streams. The presented ordination of the fish species in the mean temperature gradient will help in predicting fish community responses to climate change.

## Graphical abstract

Species–environment relationships were studied between the occurrence of 13 fish and lamprey species and 9 mainly map‐based environmental variables of Finnish boreal small streams. Modelling fish species occurrence in streams with a logistic regression and self‐organizing map indicated clear species–environment relationships. The results support the development of fish‐based bioassessment for small streams and help in predicting fish assemblage changes in a warming climate.

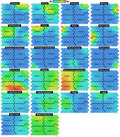

## 1 INTRODUCTION

Studying the relationship between species and their environment is at the core of ecology. Modeling this relationship has long been performed, using a wide array of methods (Domisch et al., 2015; Franklin, 1995; Guisan & Zimmermann, 2000). The focus in developing these models may be to study species–environment relationships or to predict the occurrence of the studied species. In fisheries research, the identification of the environmental variables that characterize fish distributions has been one of the main objectives (Nelson et al., 1992; Rieman & McIntyre, 1995). Predictive models may help in fish‐based bioassessment (Brosse et al., 2001; Oberdorff et al., 2001, 2002) and in focusing inventory and management activities on areas where species are considered likely to occur (Porter et al., 2000).

Several studies have indicated that field‐measured site‐scale (local) variables such as stream width, water depth, water chemistry, riverbed substrate, flowrate, undercut banks, canopy cover, riparian vegetation, and the slope at the sampling site can predict the occurrence of fish species (Gorman & Karr, 1978; Terra et al., 2016; Watson & Hillman, 1997). However, these field measurements are laborious and thus demanding for adoption as predictors of species occurrence in fisheries management, for example. An easier way to predict species occurrence would be to use large‐scale map‐based (regional) variables such as the size of the upper catchment, the elevation, and land use in the upper catchment (Porter et al., 2000). Indeed, catchment‐scale variables can have a greater impact than site‐scale variables on stream fish assemblages (DeRolph et al., 2015; Mitsuo, 2017).

The process of taking natural landscapes for human use can cause detrimental effects on terrestrial and aquatic ecosystems (Huston, 2005; Pugh et al., 2020). For example, increased land use for agriculture, urban areas, and forestry can impact fish populations through alterations in stream hydrology, geomorphology, water quality, sedimentation, riparian vegetation, and habitat heterogeneity, eventually leading to species loss or replacement (Allan et al., 1997; Lange et al., 2014; Pugh et al., 2020). Recent developments in geographical information systems (GIS) technology (Lü et al., 2019) have facilitated easy access to a wide range of catchment characteristics above any site of a stream network. These catchment characteristics, typically expressed as the percentage coverage of the upper catchment, are extensively used in studying the effects of land use on stream biota.

About 80% of the millions of kilometers of European river networks consist of small streams, commonly known as brooks, creeks, or headwaters (Kristensen & Globevnik, 2014). Small headwater streams are important contributors to aquatic biodiversity and may suppress the negative impacts of anthropogenic stress on downstream reaches (Baattrup‐Pedersen et al., 2018; Burdon et al., 2016). However, in the European Water Framework Directive (WFD; European Commission, 2000), small streams with a catchment size of <10 km^2^ are mostly omitted from river basin management plans or merged into larger water bodies (Baattrup‐Pedersen et al., 2018; Kristensen & Globevnik, 2014).

In this study, we chose to examine fish in small streams for some specific reasons. We inferred that in small streams/catchments, a single land‐use attribute such as an urban area can easily reach high coverage, and therefore, the effect of land use on fish species occurrence should be relatively easy to trace. In small streams, the upstream catchment area is always located relatively near the sampling site, and the impact of land use should therefore be more direct. Indeed, proximity to the stream has appeared an important factor in estimating the impact of land use on stream biota (Wang et al., 2001). Small streams with a small volume of water also have only a limited ability to dilute pollutants such as nutrients from agriculture (Kristensen & Globevnik, 2014). Small tributary streams have appeared to be particularly sensitive to nutrient enrichment (Bussi et al., 2018). The impact of human activities is therefore potentially greater on small water bodies than on larger ones (Kristensen & Globevnik, 2014).

Our main aims in this study were (1) to explore the relationship of map‐based environmental variables and the occurrence of fish species in small boreal streams; (2) extract fish species clusters and evaluate their ecological relevance; (3) study species occurrence in relation to annual mean temperature from the perspective of the climate change in this region; and (4) identify species–specific responses to man‐induced pressures for the future development of diagnostic indices in bioassessment of small boreal streams.

## 2 MATERIAL AND METHODS

Altogether, 11 environmental variables were measured (Table 1). The studied area covered Southern and Central Finland in the boreal region from about 60° to 67°, which are mostly covered with coniferous forest. The highest altitude among sampling sites was about 300 m in the studied territory characterized by lowlands (Table 1). The variables were map‐based, with the exception of one field‐collected variable, water temperature at sampling (electrofishing). Upstream catchment boundaries were delineated for each site with Geographical Information System, using the Digital Elevation Model (DEM) raster database from National Land Survey of Finland (NLS) and vector data of Drainage Basins in Finland (Finnish Environment Institute, SYKE). Only sites with a catchment area <100 km^2^ were included in the study. The proportions of different land covers in the catchment areas were extracted from the CORINE Land Cover 2012 data. The quantity of forest drainage by ditching was estimated as a percentage of ditched peatlands from the drainage data of the Finnish Environment Institute. Annual air temperature and precipitation data were derived from the WorldClim database (Hijmans et al., 2005).

**TABLE 1:** Basic statistics of the environmental variables studied

	Mean	Median	Min.	Max.
Latitude (°, WGS84)	62.568	62.120	60.103	66.989
Altitude (m)	98.0	84.9	1.9	305.8
Catchment area (km^2^)	16.2	9.6	0.2	98.9
Water temperature at sampling (^o^C)	11.0	11.0	1.3	21.7
Annual mean air temperature (^o^C)	2.8	3.2	−0.8	5.2
Annual precipitation (mm)	599	608	471	674
Urban areas (%)	11.0	1.7	0	78.7
Fields (%)	6.4	1.5	0	53.3
Open mires (%)	4.1	0.7	0	45.2
Lakes (%)	2.1	0.4	0	25.6
Ditched peatland (%)	9.3	7.2	0	49.0

Note

The last five variables refer to the percentages of the catchment area above the electrofishing site.

Electrofishing data from small Finnish streams were gathered mainly from a national database (Hertta/Koekalastusrekisteri) managed by the Natural Resources Institute Finland (Luke) and hosted by the SYKE. Additional data were acquired from Metsähallitus (a state‐owned enterprise responsible for the management of state‐owned land and water areas). The total number of single‐run electrofishing samples was 776, conducted at 487 sites, indicating that some of the sites were sampled more than once. As a rule, repeated sampling at the same site was performed at different years. Most of the sampling had been performed at the period 2000–2020. The electrofishing sites usually represented wadable riffles with stony bottoms. Escape nets were not used at any of the sampling sites, which typically covered 50–150 m^2^. As the electrofishing sampling had been performed in July–October, natural seasonal decline in stream water temperatures was reflected in the measured temperatures. European standard EN 14011:2003 (Water quality—sampling of fish with electricity) was followed in sampling. Fish data were converted to species presence/absence for all analyses in this study.

### 2.1 Statistical methods

The occurrence of the fish and lamprey species in relation to the environmental variables was modeled using binary logistic regression (BLR) analysis. In the preprocessing phase, highly (>0.7) multicollinear predictors (latitude and precipitation) were removed from the BLR analysis. The final number of environmental variables (predictors) accepted for BLR analyses was therefore nine (Table 1). To avoid pseudoreplication, only one randomly selected electrofishing sample per site was included (*N* = 487). Rare species, present in less than 3% of the sites, were excluded from the analysis, resulting in 13 species for the modeling (Table 2). The statistical significance of each predictor was assessed by a chi‐square test, with *p*‐value <0.05 indicating a significant impact. To assess the fit of the models to our data, Nagelkerke (pseudo) R^2^ was calculated for each model. Also Hosmer–Lemeshow goodness‐of‐fit test (Hosmer & Lemeshow, 1989) was used with *p*‐value >0.05 indicating an acceptable model fit. Accuracy of the BLR model was calculated as the percentage (%) of the studied sites where the presence or absence of a fish species was predicted correctly. BLR analyses were conducted by IBM SPSS Statistics 26.

**TABLE 2:** Goodness‐of‐fit statistics for the BLR models by fish species, predicting the probability of fish species presence (*N* = 487)

	Number of samples with fish presence	Nagelkerke *R* ^2^	Hosmer–Lemeshow test value	Accuracy (%)	Sensitivity (%)	Specificity (%)
Three‐spined stickleback	21	0.668	1.00	96.1	42.9	98.5
Ninespine stickleback	19	0.466	0.08	96.9	42.1	99.1
Perch	85	0.451	0.55	87.7	49.4	95.3
Minnow	42	0.407	0.82	92.0	11.9	99.6
Bullhead	63	0.381	0.31	89.7	36.5	97.6
Stone loach	64	0.352	0.42	88.3	23.4	98.1
Grayling	28	0.325	0.24	95.3	25.0	99.6
Roach	38	0.275	0.90	92.6	10.5	99.6
Brook trout	18	0.259	0.81	96.3	0.0	100.0
Northern pike	101	0.192	0.50	80.5	13.9	97.9
Burbot	101	0.174	0.41	79.5	8.9	97.9
Brown trout	255	0.123	0.37	62.8	72.9	51.7
Brook lamprey	28	0.058	0.08			

Note

BLR model was not statistically significant for brook lamprey.

The interactions between 13 species occurrences and 9 environmental variables were further studied using a self‐organizing map (SOM, Kohonen, 1982, 2001). In contrast to BLR, all species were processed in a single model. In general, SOM is an unsupervised dimensionality reduction method that visualizes high‐dimensional data in a low‐dimensional map. In ecology, SOM has been extensively implemented for information extraction, visualization, and clustering of community data (Chon, 2011). Compared to some conventional statistical methods (e.g., PCA, NMDS) used for community ordination, SOM has performed well, for example, by allowing the visualization of interspecific association even if it differs in different parts of the data space (Giraudel & Lek, 2001). In addition, the network tolerates noise (Vesanto et al., 1998) by allowing outlying samples to affect only one map unit and its neighborhood. The other areas of the map are not affected by these data (Kaski, 1997). In this study, unsupervised SOM was used to patternize 22 predictors (13 species + 9 environmental variables) and 487 samples with a two‐dimensional map which were then grouped, that is, clustered. This two‐stage procedure, first using SOM to produce the prototypes that are then clustered in the second stage, has been found to perform well compared with direct clustering of the data (Vesanto & Alhoniemi, 2000). The two dimensions of SOM were clustered using the k‐means algorithm (Kohonen, 2014). The Davies Bouldin validity index (Davies & Bouldin, 1979), which measures between‐ and intra‐cluster distances, was used as a performance criterion. In the parameter optimization, SOM net sizes (number of nodes in x and y dimensions) and the number of clusters in parameter k were altered, using a grid search until the minimum of the Davies Bouldin index was found, using the elbow criterion. In parameter optimization, the SOM net size roughly followed the map size rule (of thumb) of Vesanto and Alhoniemi (2000; *N*(nodes) = 5 x sqrt(N_rows_)). Each trial SOM consisted of 10,000 training rounds. In the preprocessing phase, the occurrence of each fish species was dummy (zero or one, absence or presence) coded. All predictors were then normalized with a zeroed mean and variance of one. The learning rate function was inverse of time, which ensures that all samples have an approximately equal influence on the results. The statistical analyses were performed using RapidMiner software (version Studio Large 9.7.000., https://rapidminer.com /, Mierswa et al., 2006).

## 3 RESULTS

**FIGURE 1:**
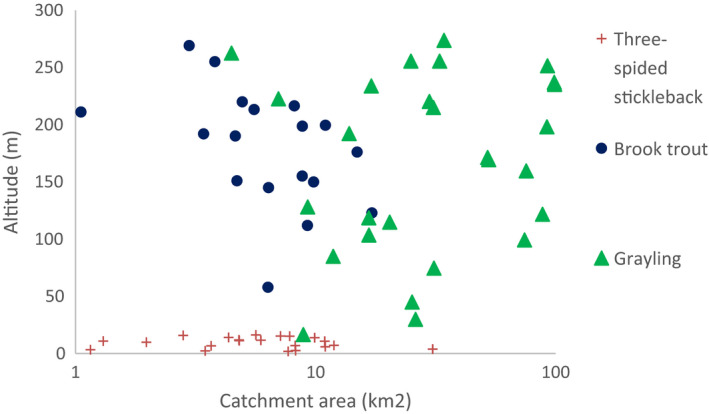
Occurrence of three fish species as a function of catchment area and altitude

As anticipated for the small catchment areas of this study, there was high variation among sites in the catchment land cover variables (Table 1). The average catchment size and altitude at the sites occupied by each of the fish species varied considerably. To illustrate this, the positioning of three species in the catchment size—altitude space suggests that three‐spined stickleback occupied small low‐altitude brooks, whereas brook trout, *Salvelinus fontinalis* (Mitchill 1814), dwelled in tributaries, and grayling, *Thymallus thymallus* L., in larger streams (Figure 1).

The BLR models were statistically significant (ꭓ^2(9)^ = 35.4 − 154.7, *p* < 0.005) for all fish species, with the exception of brook lamprey*, Lampetra planeri* (Bloch), (ꭓ^2 (9)^ = 10.1, *p* =0.340). The highest Nagelkerke *R* ^2^ values were recorded for the two stickleback species (Table 2). In Hosmer–Lemeshow goodness‐of‐fit tests, the *p*‐values were >0.08 for all the models, indicating an acceptable model fit for the data. The accuracy of the models was usually high, ranging from 96.9% with ninespine stickleback to 62.8% with brown trout. The absence of fish species was predicted by the models much more correctly than presence, as indicated by specificity (average 94.6%, *SD* 13.6%) versus sensitivity (average 28.1%, *SD* 21.1%) (Table 2).

**FIGURE 2:**
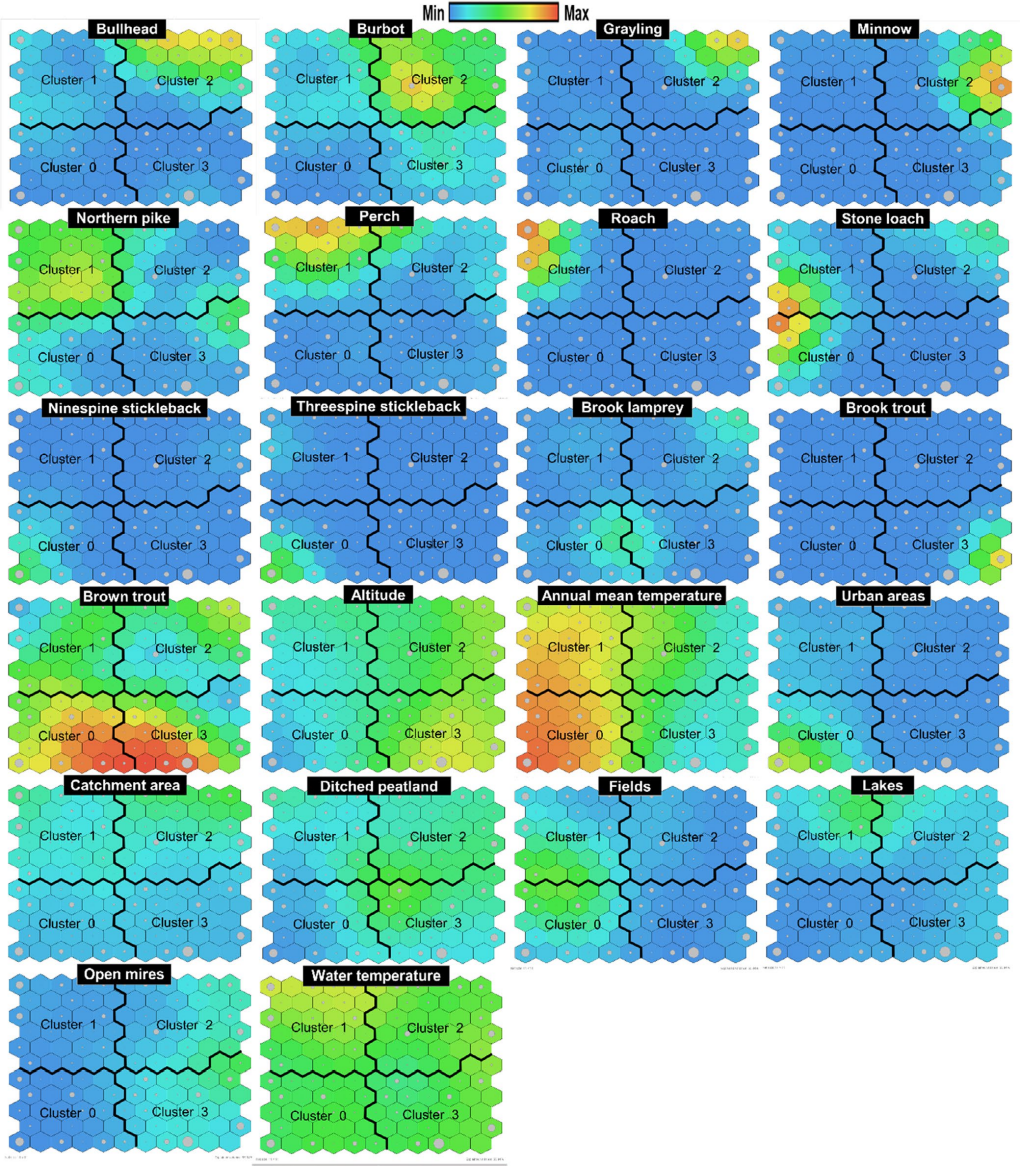
Self‐organizing maps of 22 predictors with four clusters separated by thick black lines in each figure. For example, in brook trout occurrence is highest in cluster 3, with high‐altitude sampling sites and low annual mean temperatures (see cluster 3 in the figure). Each sample (id, row) remains in the same SOM node (cell) in each figure. The sample size of brown trout (255, Table 2) was higher than that of grayling (28, Table 2), and hence, the general coloring of grayling figure in the topmost row is bluer. The size of gray circles represents the number of samples in a cell

The statistical performance of the best SOM model including all species was good (explained variance 55.94%, Davies Bouldin index 0.71). The net size (11 × 11 = 121 nodes) of the best‐performing SOM model roughly followed the map size rule of thumb (110 nodes). The smallest Davies Bouldin index was attained with 4 clusters (Figure 2). Cluster 2 at the top right of the SOM was occupied by bullhead, burbot, *Lota lota* (L.), grayling, and minnow, *Phoxinus phoxinus* (L.), and characterized by a large catchment area, high altitude, and low mean temperature (Figure 2, Table 3). Cluster 1 at the top left of the SOM was occupied by perch, *Perca fluviatilis* L., roach, *Rutilus rutilus* (L.), and northern pike, *Esox* *Lucius* L. and characterized by a high water temperature at sampling, high annual mean temperature, large catchment area, and low altitude (Figure 2, Table 3). Cluster 0 at the bottom left of the SOM was occupied by the two stickleback species, and characterized by a low altitude, high annual mean temperature, low water temperature at sampling, and high percentage of urban areas in the catchment. Brook trout was present in sites clustered at the bottom right of the SOM, indicating preference for cold high‐altitude tributaries. Brown trout and stone loach, *Barbatula barbatula* (L.), seemed to occupy two clusters simultaneously, whereas the occurrence of brook lamprey could not be linked with any of the studied environmental variables (Table 3, Figure 2).

**TABLE 3:** Significance (*p*) values from the logistic regression analysis run separately for each fish species–predictor pair

	Catchment area	Altitude	Water temperature	Annual mean temperature	Fields	Ditched peatland	Urban areas	Open mires	Lakes	Cluster
Bullhead	**<0.001**	**0.043**		*0.001*			*0.024*	**0.020**	**0.003**	2
Burbot	**<0.001**	**0.001**	**0.001**	*<0.001*	*0.035*		*0.003*	**0.001**	**0.023**	2
Grayling	**<0.001**	**<0.001**		*<0.001*				**0.001**		2
Minnow	**<0.001**	**<0.001**	**0.012**	*<0.001*	*0.004*	**0.001**	*0.001*	**<0.001**		2
Northern pike	**0.011**	*<0.001*	**0.003**	**<0.001**	**0.002**				**0.024**	1
Perch	**<0.001**	*0.004*	**<0.001**	**0.003**					**<0.001**	1
Roach	**<0.001**	*0.002*	**<0.001**	**0.002**				*0.011*		1
Stone loach	**0.008**	*<0.001*		**<0.001**	**<0.001**	*<0.001*		*0.001*	*0.019*	0 ~ 1
Ninespine stickleback		*<0.001*	*0.012*	**0.001**		*0.003*	**<0.001**	*0.033*		0
Three‐spined stickleback	*0.019*	*<0.001*	*0.011*	**<0.001**		*<0.001*	**<0.001**	*0.011*		0
Brook lamprey										‐
Brook trout	*0.031*	**0.001**		*0.001*						3
Brown trout			*<0.001*			*0.002*				0 ~ 3

Notes

**FIGURE 3:**
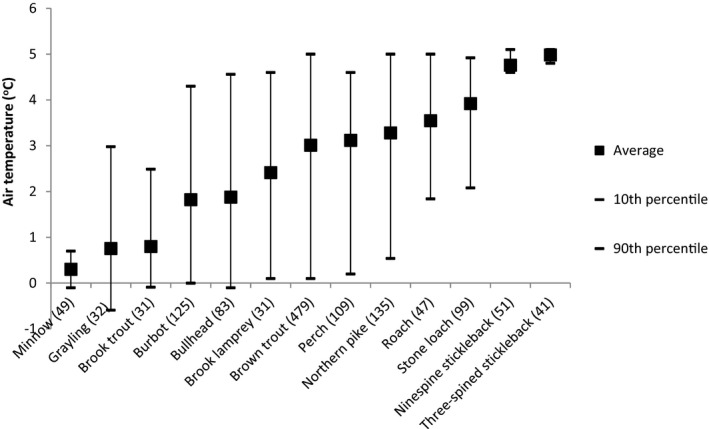
Annual average air temperature at the sites where each of the species occurred (*N* in parenthesis)

Values in bold indicate positive effect (*p* < 0.05), values in italics negative effect (*p* < 0.05), in missing values *p* > 0.05. Clusters 0–3 refer to the SOM analysis results presented in Figure 3.

The ranking of species in the mean air temperature gradient revealed the two stickleback species favored a warm environment, whereas minnow appeared to be the ultimate cold‐water species (Figure 3).

## 4 DISCUSSION

Modeling fish species occurrence in small boreal streams with a logistic regression and self‐organizing map indicated clear species–environment relationships. The obtained species clusters and their associations with mainly map‐based variables appeared ecologically reasonable and largely concordant with species groupings in the current bioassessment developed for larger boreal streams (Vehanen et al., 2010). The results support the development of fish‐based bioassessment for small streams and help in predicting fish assemblage changes in a warming climate.

The effect of small‐scale local factors on controlling the occurrence of lotic fish species has been found in numerous studies (Lamouroux et al., 1999; Wang et al., 2003; Watson & Hillman, 1997). However, the dominance of large‐scale regional factors affecting riverine fish assemblages has also been documented (DeRolph et al., 2015; Koel & Peterka, 2003; Mitsuo, 2017). A wide variety of hypotheses or theories has been put forward concerning the balance of local and regional factors affecting riverine fish assemblages. It has been hypothesized that large‐scale processes determine the pool of the fish species available to occur, whereas small‐scale processes eventually define the subset of fish species inhabiting a given site (Pont et al., 2005). Although local habitat conditions may be important determinants of fish abundance, they may be of limited importance in determining presence and absence (Porter et al., 2000). Sensitivity to local‐ and regional‐scale processes has been found species‐specific (Pont et al., 2005). It was suggested that local factors were most important to fish in minimally impaired watersheds, but the effects of landscape‐scale factors become increasingly important as watersheds are increasingly modified by human activities (Wang et al., 2003). However, a combination of local and regional variables has often managed to explain a great deal of the variance in riverine fish occurrence or density (Park et al., 2006; Pont et al., 2005; Ripley et al., 2005). Obviously, both local and regional variables have an effect, and the inclusion of local variables in our models would probably have enhanced the predictive power. However, our results encourage the use of map‐based (regional) variables in modeling the species–environment relationships in small streams, especially when confronting limited resources to control site‐specific local variables.

The sensitivity of the BLR model was rather poor, at least compared to specificity (Table 2). The relatively small size of the electrofishing area and the use of a single‐run electrofishing sampling in this study may have decreased the probability of getting all the fish species in the catch. The information generated by single‐visit surveys of fish occurrence cannot account for intra‐annual or interannual variation in the upstream extent of fish distribution (Fransen et al., 2006). Small streams are vulnerable to drought events inducing temporal variation in fish assemblages (Grossman et al., 1998; Keaton et al., 2005). Our model's prediction of species occurrence and absence may be of use in extending the current fish‐based bioassessment (Vehanen et al., 2010) to small brooks. For management and inventory purposes, we recommend the application of larger data and cross‐validation in BLR.

The SOM clusters of fish species and environmental variables appeared plausible. Cluster 0 was occupied by two stickleback species that seemed to favor warm regions, low altitude, and the high share of urban areas in the upper catchment. Sticklebacks have been considered to indicate degradation in lowland brooks (Fieseler & Wolter, 2006). Freshwater fish communities have been found sensitive to watershed urbanization (Chen & Olden, 2020).

The occurrence of perch, roach, and northern pike (Cluster 1 in SOM) was associated with a high annual mean temperature, a relatively large catchment area, low altitude, and lakes in the upper catchment. These three fish species are common lake species (Maitland & Campbell, 1992) possibly spreading to small streams at warm‐water periods (Degerman & Sers, 1994; Sutela et al., 2017). This trait was supported by the frequent occurrence of these species with high temperature at sampling (Table 3). The occurrence of bullhead, burbot, grayling, and minnow was associated with a relatively large catchment area, a high altitude, a low mean temperature, and open mires in the catchment (Cluster 2). The fish species in this cluster can be characterized as cold‐water species (Logez et al., 2012) living in forested peatland regions. The only fish species centering cluster 3, brook trout, favored cold, and small high‐altitude streams. Brook trout is an alien invader species in Europe, having been stocked in many Finnish tributary streams. Brook trout also prefers small tributary streams in its home district in North America (Kanno et al., 2015). Alien brook trout has been found to exclude brown trout in small Finnish brooks (Korsu et al., 2007).

The appearance of the most frequently encountered fish species, brown trout, was centered in clusters 0 and 3 with avoidance of ditched peatland in the upper catchment. The drainage ditching of peatland for forestry causes the erosion and deposition of fine sediments in headwater streams, accompanied by nutrient loading (Marttila & Kløve, 2010; Nieminen et al., 2018). Deposited sediment can diminish salmonid embryo survival by decreasing redd gravel permeability, interstitial water exchange, and therefore oxygen supply (Greig et al., 2007; Louhi et al., 2011; Michel et al., 2014). These impacts may have suppressed the occurrence of brown trout in catchments with a high coverage of ditched peatland in this study.

Climate change scenarios forecast a high increase in the mean air temperature for the European boreal ecoregion (Schneider et al., 2013). Fish species have evolved to fit distinct thermal niches where they can optimize physiological, reproductive, and ecological performance (Coutant, 1987; Graham & Harrod, 2009). Temperature is one of the key abiotic factors affecting fish species distribution (Matthews, 1998). Globally, fish species living in small headwater streams are especially vulnerable to climate change (Buisson & Grenouillet, 2009; Buisson et al., 2008). The presented ranking of the fish species along the mean air temperature gradient can help in predicting the effects of a warming climate on fish assemblages in the studied region. The breadth of the thermal range largely delineates the ability of fish species to adapt to climate change (Buisson & Grenouillet, 2009; Logez et al., 2012). In this study, minnow expressed a relatively narrow thermal range at the cold end of the gradient (Figure 3), suggesting high vulnerability to the warming climate in this region. The thermal ranges of some fish species (e.g., brown trout, perch, and northern pike) may vary, depending on the size of the catchment area and stream power (Logez et al., 2012). Accordingly, the inclusion of large rivers in the analyses could result in a different outcome for fish species ordination along the mean temperature gradient. These findings suggest that local stream characteristics should be taken into account when predicting the effects of climate change. Besides the increase in the mean air and river water temperature in the European boreal ecoregion, future winter discharges are likely to increase from the natural flow regime, while summer flows will be less impacted (Schneider et al., 2013). The discharge aspect, although probably of minor importance in the boreal region, should also be taken into account when predicting the effects of a warming climate on boreal riverine fish assemblages.

The assessment of the ecological status or integrity of surface waters has been widely established around the world (Karr & Chu, 2000; Poikane et al., 2020; Xu et al., 2014). In Europe, the legislation to achieve a good ecological status in surface waters is guided by the WFD (European Commission, 2000). Bioassessment methods in rivers have been developed using three biological groups: periphytic diatoms, benthic invertebrates, and fish fauna. Stream biota is often impaired by multiple pressures interacting in additive, synergistic, or antagonistic ways (Schinegger et al., 2012). Diagnostic tools for distinguishing the impacts of different pressures have been called for to target the diminishing measures in water pollution control (Lemm et al., 2019; Poikane et al., 2020). In this study, map‐derived pressures of agriculture (fields), urban land cover, and drainage ditching for forestry seemed to affect the occurrence of certain fish species. These results encourage the development of diagnostic fish‐based pressure‐specific metrics for small boreal streams.

A simple diagnostic tool (index) for evaluating direct effects of climate change could be calculated as an average of two metrics, the proportion of cold‐water species (climate change intolerants, scaled to 0–1), and the proportion of warm‐water species (climate change tolerants, scaled to 0–1, inverse values) of an electrofishing sample. Referring to Figure 3, in our case the cold‐water species could be minnow, grayling and brook trout, and the warm‐water species three‐spined stickleback, ninespine stickleback, and stone loach. For a wider use of this index, temperature preferences could be achieved like in this study or by using existing knowledge and references about temperature preferences of fish species, such as Logez et al. (2012). Possible indirect effects of climate change stemming from flushing of nutrients (Wilby et al., 2006), for instance, could be integrated to the index following the basics presented in Hering et al. (2006).

In the fish‐based integrity indices developed in bioassessment for boreal and northern temperate zone, cool‐ or cold‐water fish species are often classified as intolerant species (Kanno et al., 2010; Vehanen et al., 2010). This feature is also seen in the Figure 3, where seven species from the left indicating favor of cold water can be classified as intolerant (grayling, brook trout, bullhead, brook lamprey, and brown trout) or intermediately tolerant (minnow and burbot) referring to Holzer (2008). Respectively, the six species on the right‐hand side indicating favor of warm water can be classified as tolerant (perch, roach, ninespine stickleback, and three‐spined stickleback) or intermediately tolerant (northern pike and stone loach). The classification of tolerant and intolerant species by Holzer is highly compatible with those used in fish integrity indices, such as Pont et al. (2006), Hughes et al. (2004), Vile and Henning (2018), and Vehanen et al. (2010). Observed pattern in the sequence of species in the temperature gradient in relation to tolerant–intolerant division of species suggests that the integrity indices developed for regions inhabited by these species should respond to the water temperature rise in streams on itself, without the possible influence via indirect effects such as altered discharge regime and flushing of extra nutrients (Wilby et al., 2006). In other words, the effect of climate change strictly as warming of the streams should be (by chance) at least to some extent inborn in many of the present fish indices. As an example, cool‐water versus warm‐water species balance obviously affects the FiFI index values, which can be easily approximated or calculated based on the metrics by Vehanen et al. (2010). At any rate, when aiming to integrate the effect of global warming to fish indices, the effect of warming on the reference sites should be controlled by referring to the earliest reliable electrofishing data or other historical fish data. This somewhat different approach from the more adaptable attitude to the direct effects of climate change in WFD (Kristensen et al., 2018; Nõges et al., 2007) could be considered also with other biological quality elements.

## CONFLICT OF INTEREST

The authors declare no conflict of interest.

## AUTHOR CONTRIBUTION

**Tapio Sutela:** Writing‐original draft (lead). **Teppo Vehanen:** Methodology (supporting); Writing‐original draft (supporting). **Pekka Jounela:** Methodology (lead); Writing‐original draft (supporting). **Jukka Aroviita:** Methodology (supporting); Writing‐original draft (supporting).

## Data Availability

Data are available from the Dryad Digital Repository (https://doi.org/10.5061/dryad.6t1g1jwzp).
